# Pharmacokinetic studies for proving bioequivalence of orally inhaled drug products—critical issues and concepts

**DOI:** 10.3389/fphar.2015.00117

**Published:** 2015-06-03

**Authors:** Karan Thakkar, Suyog Mhatre, Manish Jadhav, Sailendra Goswami, Rajen Shah

**Affiliations:** Raptim Research Ltd.Mumbai, India

**Keywords:** inhalation, bioequivalence studies, metered dose inhalers, dry powder inhalers, peak flowmeter, spacer, charcoal block

The inhalational drug market, especially the generic market, has a tremendous growth potential globally (GBI Research, 2011; Espicom, 2013; Transperancy: Market Research, 2013). Generics are drugs that are bioequivalent to the approved drugs. The United States Food and Drug Administration (FDA) has defined bioequivalence as (U.S. FDA, 2014), “The absence of a significant difference in the rate and extent to which the active ingredient or active moiety in pharmaceutical equivalents or pharmaceutical alternatives becomes available at the site of drug action when administered at the same molar dose under similar conditions in an appropriately designed study.”

The inhalational route of drug delivery has various advantages (Morgan et al., 1986; Rau, 2005):

1. Delivery of the drugs directly at the site of action.
2. Faster onset of action.
3. Lower systemic concentration and hence lesser adverse drug reactions.
4. Absence of first pass metabolism in m ay cases, permitting use of a lower dose in the formulation; and more reliable and predictable action.

Generally, to prove bioequivalence of orally inhaled drug products (OIDPs), *in-vitro* and *in-vivo*–pharmacokinetic (PK), and dynamic (PD) studies are required (Committee For Medicinal Products For Human Use, 2009; Daley-Yates and Parkins, 2011; Lee, 2011; Office of Generic Drugs, 2013). Currently, there is a considerable amount of ongoing debate regarding the universally acceptable methodology for conducting PK studies for inhaled drugs (Daley-Yates and Parkins, 2011). Nonetheless, PK studies to prove bioequivalence of inhaled drugs play a very important role toward the goal of ensuring substitutability of generics, especially when *in-vitro* data and pharmaceutical data are conflicting (O'Connor et al., 2011).

There has been a lot of interest and discussion among the pharmaceutical companies, regulators, and academia with regards to bioequivalence studies of OIDPs (Hochhaus et al., 2015). In the conference co-organized by the University of Florida and the International Pharmaceutical Aerosol Consortium on Regulation and Science (IPAC-RS) held in March 2014, the main points discussed were: subject selection for PK studies of OIDPs, PK study design, *in-vitro* and PK correlations, regulatory views and so on (Hochhaus et al., 2015). With more and more pharmaceutical companies wanting to introduce generic OIDPs, it has become imperative to understand certain key concepts involved in conducting the PK studies for OIDPs.

There exist certain critical issues in conducting the PK studies for proving bioequivalence of inhaled drugs:

1. *Dose selection:* Since the inhalational route delivers the drug at the site of action, the systemic concentration is very low, sometimes too low to be detected by the standard bioanalytical methods. This either requires increasing the dose of the drug or developing more sensitive methods of drug assay (Silvestro et al., 2012). Increasing the dose could endanger the safety of human volunteers, for example, increased incidence of tremors, palpitations and hypokalemia due to Salbutamol (Lipworth et al., 1989; Fowler and Lipworth, 2001), anticholinergic side effects due to Glycopyrronium and Tiotropium (Durham, 2004; Hansel et al., 2005; Loke and Singh, 2013), etc.
2. *Subject selection:* Healthy and non-smoking volunteers are to be selected for the PK study. The reasons for including non-smokers are ((US) Office on Smoking and Health, 2006; Gold et al., 1996):
  - Smokers are more liable to have respiratory morbidities which may affect the comparative pharmacokinetics (Zarowitz et al., 1985; Sjosward et al., 2003),
  - Smoking leads to induction of various metabolic enzymes like CYP 1A1 and 1B1 (Kroon, 2007; Olsson et al., 2011), and
  - Smokers have an altered muco-ciliary clearance and local microenvironment (Scott, 2004).

These factors may introduce intra-subject variability even in cross-over studies since the cumulative effect of a combination of these factors may vary in the same individual at different times. Smokers can be detected objectively by conducting a urine cotinine test and excluded from the study (Parker et al., 2002; Jung et al., 2012).

Volunteers can be screened for respiratory diseases by conducting medical history and examination, chest x-rays and pulmonary function testing (PFT). PFT can be conducted using Spirometers or more preferably, Peak Flow Meters (PFM), especially in the non-hospital settings (Quanjer et al., 1997). Studies have reported that the PFM may slightly over estimate the expiratory flow rate, but the difference was not found to be significant (Quanjer et al., 1997; Gupta and Agarwal, 2007). Nonetheless, proper procedure for testing with PFM should be followed, like (Quanjer et al., 1997): application of nose-clip, asking the subject to form a tight seal around the mouthpiece of the PFM with their lips and to exhale as forcefully, rapidly and completely as possible in about 1–2 s. The test may be repeated for a minimum of three times but no more than eight times and the gap between the end of maximum inhalation and the beginning of maximal rapid exhalation should not be more than 2 s (Quanjer et al., 1997).

3. *Subject training:* This is one of the most important factors for assuring proper performance of the PFM testing and, more importantly, for correct and consistent inhalation technique (Leiva-Fernández et al., 2012; Göriş et al., 2013; Rahmati et al., 2014). The key points to be emphasized while training for correct inhalation technique are:
  - Complete exhalation before beginning of inhalation.
  - Ensuring a firm seal with the lips around the device mouth piece.
  - For Metered Dose Inhaler (Göriş et al., 2013): The most important thing is co-ordination of actuation and inhalation. After complete exhalation, the subject should be asked to breathe in slowly and deeply for 5–10 s. The device should be actuated while the inhalation is going on. After this the subject should be asked to hold his/her breath for 5–10 s and then breathe out normally through the nose.
  - For Dry Powder inhalers (Chrystyn, 2007; Lavorini et al., 2008): Here, the energy for propelling and inhaling the drug is provided by the individual, that is, they are breath actuated. Hence, the inhalation attempt has to be rapid and deep with quick acceleration over 3–4 s. After this the subject should be asked to hold his/her breath for 5–10 s and then breathe out normally through the nose. The most common mistake while using a DPI is not completely exhaling before beginning of inhalation followed by not holding breath adequately.

Various aids and instruments are available for inhalation training of patients/ volunteers (Al-Showair et al., 2007; Lavorini et al., 2010; Yawn et al., 2012; Lavorini, 2013).

4. *Other factors:*
  - *Use of a spacer with an MDI:* Using a spacer an MDI obviates the need for coordination between inhalation and actuation and also decreases the deposition of the drug particles in the oropharynx (Lavorini and Fontana, 2009). As per the European Agency for Evaluation of Medicinal Products (EAM) (Committee For Proprietary Medicinal Products, 2004), if a product has been licensed for use only with a spacer, the PK studies should have a spacer. If the product can be used with or without a spacer, two PK studies would be required, one with and the other without the spacer. As per our personal communication with a consultant regarding the U.S. FDA, 2014 requirements, spacers are not required for PK studies unless the product is to be used only with spacers.
  - *Use of charcoal block (Adams et al., 2010)*: Administering charcoal suspension at various intervals eliminates the enteral absorption of the proportion of inhaled drug which may be swallowed and the systemic concentration reflects only that fraction of the drug that is absorbed form the respiratory tract. Use of charcoal block is not mandatory for PK studies for regulatory submissions, except for EMA submissions (Lu et al., 2015). If used, the method of administering charcoal should be adequately validated. EMA requires two studies: one with and one without charcoal block (Lu et al., 2015).

To summarize, the following are recommended:

1. Using the least possible dose and developing sensitive bioanalytical methods.
2. Selection of healthy and non-smoking volunteers. Their smoking status can be judged by the urine cotinine test. Their screening PFT can be done by using PFM as described above.
3. Subject training for correct performance of the PFM test and inhalation of drug from the MDI/DPI is the most important aspect. For MDI, steady and gentle inhalation in coordination with actuation and for DPI rapid, forceful and deep inhalation is required.
4. Spacers may be used with MDIs if required. If charcoal block is used, the procedure should be adequately validated.

## Conflict of interest statement

The authors declare that the research was conducted in the absence of any commercial or financial relationships that could be construed as a potential conflict of interest.

## References

[B1] Adams W. P. Ahrens R. C. Chen M.-L. Christopher D. Chowdhury B. A. Conner D. P.. (2010). Demonstrating bioequivalence of locally acting orally inhaled drug products (OIPs): workshop summary report. J. Aerosol Med. Pulm. Drug Deliv. 23, 1–29. 10.1089/jamp.2009.0803 20131983

[B2] Al-Showair R. A. M. Pearson S. B. Chrystyn H. (2007). The potential of a 2Tone Trainer to help patients use their metered-dose inhalers. Chest 131, 1776–1782. 10.1378/chest.06-2765 17400675

[B3] Chrystyn H. (2007). The Diskus: a review of its position among dry powder inhaler devices. Int. J. Clin. Pract. 61, 1022–1036. 10.1111/j.1742-1241.2007.01382.x 17504364 PMC1974824

[B4] Committee For Medicinal Products For Human Use. (2009). Guideline on The Requirements for Clinical Documentation for Orally Inhaled Products (Oip) Including the Requirements for Demonstration of Therapeutic Equivalence between Two Inhaled Products for Use in the Treatment of Asthma and Chronic Obstructive Pulm. European Medicines Agency. Available online at: http://www.ema.europa.eu/docs/en_GB/document_library/Scientific_guideline/2009/09/WC500003504.pdf

[B5] Committee For Proprietary Medicinal Products. (2004). Points to Consider on the Requirements for Clinical Documentation for Orally Inhaled Products (Oip). Available online at: http://www.ema.europa.eu/docs/en_GB/document_library/Scientific_guideline/2009/09/WC500003558.pdf

[B6] Daley-Yates P. T. Parkins D. A. (2011). Establishing bioequivalence for inhaled drugs; weighing the evidence. Expert Opin. Drug Deliv. 8, 1297–1308. 10.1517/17425247.2011.592827 21699442

[B7] Durham M. C. (2004). Tiotropium (Spiriva): a once-daily inhaled anticholinergic medication for chronic obstructive pulmonary disease. Proc. (Bayl. Univ. Med. Cent). 17, 366–373. 16200123 10.1080/08998280.2004.11927996 PMC1200675

[B8] Espicom. (2013). Inhalation & nasal spray generic drugs 2013. Bus. Monit. Int. Available online at: http://www.espicom.com/inhalation-nasal-spray-generic-drugs-2013.html

[B9] Fowler S. J. Lipworth B. J. (2001). Pharmacokinetics and systemic beta2-adrenoceptor-mediated responses to inhaled salbutamol. Br. J. Clin. Pharmacol. 51, 359–62. 10.1046/j.1365-2125.2001.01362.x 11318774 PMC2014458

[B10] GBI Research. (2011). Drug delivery device market to 2017 – metered dose inhalers and infusion pumps to be key revenue generators. Glob. Bus. Inteliigence. Available online at: http://www.gbiresearch.com/report-store/market-reports/medtech/drug-delivery-devices-to-2017-metered-dose-inhalers-and-infusion-pumps-to-be-key-revenue-generators/send-to-friend

[B11] Gold D. R. Wang X. Wypij D. Speizer F. E. Ware J. H. Dockery D. W. (1996). Effects of cigarette smoking on lung function in adolescent boys and girls. N. Engl. J. Med. 335, 931–937. 10.1056/NEJM199609263351304 8782500

[B12] Göriş S. Taşci S. Elmali F. (2013). The effects of training on inhaler technique and quality of life in patients with COPD. J. Aerosol Med. Pulm. Drug Deliv. 26, 336–344. 10.1089/jamp.2012.1017 23421900

[B13] Gupta P. Agarwal D. (2007). A comparison of peak expiratory flow measured from forced vital capacity and peak flow meter manoeuvres in healthy volunteers. Ann. Thorac. Med. 2, 103. 10.4103/1817-1737.33697 19727355 PMC2732084

[B14] Hansel T. T. Neighbour H. Erin E. M. Tan A. J. Tennant R. C. Maus J. G.. (2005). Glycopyrrolate causes prolonged bronchoprotection and bronchodilatation in patients with asthma. Chest 128, 1974–1979. 10.1378/chest.128.4.1974 16236844

[B15] Hochhaus G. Horhota S. Hendeles L. Suarez S. Rebello J. (2015). Pharmacokinetics of orally inhaled drug products. AAPS J. 17, 769–775. 10.1208/s12248-015-9736-6 25762449 PMC4406971

[B16] Jung S. Lee I. S. Kim S. B. Moon C. S. Jung J. Y. Kang Y. A.. (2012). Urine cotinine for assessing tobacco smoke exposure in Korean: analysis of the Korea National Health and Nutrition Examination Survey (KNHANES). Tuberc. Respir. Dis. (Seoul). 73, 210–218. 10.4046/trd.2012.73.4.210 23166556 PMC3492421

[B17] Kroon L. A. (2007). Drug interactions with smoking. Am. J. Health Syst. Pharm. 64, 1917–1921. 10.2146/ajhp060414 17823102

[B18] Lavorini F. (2013). The challenge of delivering therapeutic aerosols to asthma patients. ISRN Allergy 2013, 102418. 10.1155/2013/102418 23984095 PMC3747606

[B19] Lavorini F. Fontana G. A. (2009). Targeting drugs to the airways: the role of spacer devices. Expert Opin. Drug Deliv. 6, 91–102. 10.1517/17425240802637862 19236210

[B20] Lavorini F. Levy M. L. Corrigan C. Crompton G. (2010). The ADMIT series - issues in inhalation therapy. 6) training tools for inhalation devices. Prim. Care Respir. J. 19, 335–341. 10.4104/pcrj.2010.00065 21049263 PMC6602267

[B21] Lavorini F. Magnan A. Dubus J. C. Voshaar T. Corbetta L. Broeders M.. (2008). Effect of incorrect use of dry powder inhalers on management of patients with asthma and COPD. Respir. Med. 102, 593–604. 10.1016/j.rmed.2007.11.003 18083019

[B22] Lee S. (2011). Scientific and Regulatory Considerations for Bioequivalence (BE) of Dry Powder Inhalers (DPIs). Available online at: http://www.fda.gov/downloads/Drugs/DevelopmentApprovalProcess/HowDrugsareDevelopedandApproved/ApprovalApplications/AbbreviatedNewDrugApplicationANDAGenerics/UCM292652.pdf

[B23] Leiva-Fernández F. Leiva-Fernández J. Zubeldia-Santoyo F. García-Ruiz A. Prados-Torres D. Barnestein-Fonseca P. (2012). Efficacy of two educational interventions about inhalation techniques in patients with chronic obstructive pulmonary disease (COPD). *TECEPOC:* study protocol for a partially randomized controlled trial (preference trial). Trials 13, 64. 10.1186/1745-6215-13-64 22613015 PMC3404981

[B24] Lipworth B. J. Brown R. A. McDevitt D. G. (1989). Assessment of airways, tremor and chronotropic responses to inhaled salbutamol in the quantification of beta 2-adrenoceptor blockade. Br. J. Clin. Pharmacol. 28, 95–102. 10.1111/j.1365-2125.1989.tb03510.x 2570601 PMC1379975

[B25] Loke Y. K. Singh S. (2013). Risk of acute urinary retention associated with inhaled anticholinergics in patients with chronic obstructive lung disease: systematic review. Ther. Adv. Drug Saf. 4, 19–26. 10.1177/2042098612472928 25083248 PMC4110819

[B26] Lu D. Lee S. L. Lionberger R. A. Choi S. Adams W. Caramenico H. N.. (2015). International guidelines for bioequivalence of locally acting orally inhaled drug products: similarities and differences. AAPS J. 17, 546–557. 10.1208/s12248-015-9733-9 25758352 PMC4406956

[B27] Morgan D. J. Paull J. D. Richmond B. H. Wilson-Evered E. Ziccone S. P. (1986). Pharmacokinetics of intravenous and oral salbutamol and its sulphate conjugate. Br. J. Clin. Pharmacol. 22, 587–93. 10.1111/j.1365-2125.1986.tb02939.x 3790406 PMC1401185

[B28] O'Connor D. Adams W. P. Chen M.-L. Daley-Yates P. Davis J. Derendorf H.. (2011). Role of pharmacokinetics in establishing bioequivalence for orally inhaled drug products: workshop summary report. J. Aerosol Med. Pulm. Drug Deliv. 24, 119–135. 10.1089/jamp.2011.0878 21453049

[B29] Office of Generic Drugs. (2013). Draft Guidance on Albuterol Sulfate. Available online at: http://www.fda.gov/downloads/Drugs/GuidanceComplianceRegulatoryInformation/Guidances/UCM346985.pdf

[B30] Olsson B. Bondesson E. Borgström L. Edsbäcker S. Eirefelt S. Ekelund K. (2011). Pulmonary drug metabolism, in Controlled Pulmonary Drug Delivery, eds Smyth H. D. C. Hickey A. J. (New York, NY: Springer), 21–50. 10.1007/978-1-4419-9745-6_2

[B31] Parker D. R. Windsor R. Wilkins J. Heimdal J. Lasater T. M. Upegui D. I. (2002). The accuracy of self-reported smoking status assessed by cotinine test strips. Nicotine Tob. Res. 4, 305–309. 10.1080/14622200210142715 12215239

[B32] Quanjer P. H. Lebowitz M. D. Gregg I. Miller M. R. Pedersen O. F. (1997). Peak expiratory flow: conclusions and recommendations of a Working Party of the European Respiratory Society. Eur. Respir. J. Suppl. 24, 2S–8S. 9098701

[B33] Rahmati H. Ansarfard F. Ghodsbin F. Ghayumi M. A. Sayadi M. (2014). The effect of training inhalation technique with or without spacer on maximum expiratory flow rate and inhaler usage skills in asthmatic patients: a randomized controlled trial. Int. J. Community Based Nurs. Midwifery 2, 211–219. 25349864 PMC4201214

[B34] Rau J. L. (2005). The inhalation of drugs: advantages and problems. Respir Care 50, 367–382. 15737247

[B35] Scott J. E. (2004). The pulmonary surfactant: impact of tobacco smoke and related compounds on surfactant and lung development. Tob. Induc. Dis. 2:1. 10.1186/1617-9625-2-1 19570267 PMC2671518

[B36] Silvestro L. Savu S. R. Savu S. N. Tudoroniu A. Tarcomnicu I. (2012). Development of a sensitive method for simultaneous determination of fluticasone propionate and salmeterol in plasma samples by liquid chromatography-tandem mass spectrometry. Biomed. Chromatogr. 26, 627–635. 10.1002/bmc.1708 22577660

[B37] Sjosward K. N. Josefsson M. Ahlner J. Andersson R. G. G. Schmekel B. (2003). Metabolism of salbutamol differs between asthmatic patients and healthy volunteers. Pharmacol. Toxicol. 92, 27–32. 10.1034/j.1600-0773.2003.920105.x 12710594

[B38] Transperancy: Market Research. (2013). Inhalation & nasal spray generic drugs market – global industry analysis, size, share, growth, trends and forecast, 2013–2019. Transperancy Mark. Res. Available online at: http://www.transparencymarketresearch.com/inhalation-nasal-spray-generic-drugs.html

[B39] U.S. FDA. (2014). Bioavailability And Bioequivalence Requirements. United States of America. Available online at: http://www.accessdata.fda.gov/scripts/cdrh/cfdocs/cfcfr/CFRSearch.cfm?CFRPart=320&showFR=1&subpartNode=21:5.0.1.1.8.1

[B40] (US) Office on Smoking and Health. (2006). Introduction, Summary, and Conclusions. Available online at: http://www.ncbi.nlm.nih.gov/books/NBK44328/ (Accessed April 6, 2015)

[B41] Yawn B. P. Colice G. L. Hodder R. (2012). Practical aspects of inhaler use in the management of chronic obstructive pulmonary disease in the primary care setting. Int. J. Chron. Obstruct. Pulmon. Dis. 7, 495–502. 10.2147/COPD.S32674 22888221 PMC3413176

[B42] Zarowitz B. Shlom J. Eichenhorn M. S. Popovich J. (1985). Alterations in theophyllineprotein binding in acutely ill patients with COPD. CHEST J. 87, 766-769. 10.1378/chest.87.6.766 3996064

